# The Impact of PI3-kinase/RAS Pathway Cooperating Mutations in the Evolution of *KMT2A*-rearranged Leukemia

**DOI:** 10.1097/HS9.0000000000000195

**Published:** 2019-04-17

**Authors:** Maria Teresa Esposito

**Affiliations:** Department of Life Sciences, Health Science Research Center, University of Roehampton, Whiteland College, London.

## Abstract

Leukemia is an evolutionary disease and evolves by the accrual of mutations within a clone. Those mutations that are systematically found in all the patients affected by a certain leukemia are called “drivers” as they are necessary to drive the development of leukemia. Those ones that accumulate over time but are different from patient to patient and, therefore, are not essential for leukemia development are called “passengers.” The first studies highlighting a potential cooperating role of phosphatidylinositol 3-kinase (PI3K)/RAS pathway mutations in the phenotype of *KMT2A*-rearranged leukemia was published 20 years ago. The recent development in more sensitive sequencing technologies has contributed to clarify the contribution of these mutations to the evolution of *KMT2A*-rearranged leukemia and suggested that these mutations might confer clonal fitness and enhance the evolvability of *KMT2A*-leukemic cells. This is of particular interest since this pathway can be targeted offering potential novel therapeutic strategies to *KMT2A*-leukemic patients. This review summarizes the recent progress on our understanding of the role of PI3K/RAS pathway mutations in initiation, maintenance, and relapse of *KMT2A*-rearranged leukemia.

## Abstract 2

Supplemental Digital Content is available in the text

## Background

Many cancers are the result of 2 or multiple sequential hits that damage the DNA.^1^ In the 2-hit model of leukemia, the initial genetic hit often leads to abnormal cell differentiation (Type II mutation) (such as *PML-RARα*, *AML1-ETO*, *KMT2A* rearrangements) while subsequent mutations may activate specific signaling pathways that are involved in cell proliferation, such as *FLT3*, *c-Kit* and the RAS/MAP kinase pathway (Type I mutation).^2^ Chromosomal translocations affecting the *Histone-Lysine-N-methyl-transferase 2A* gene (*KMT2A*, previously known as Mixed Lineage Leukemia, MLL) at 11q23 are a genetic hallmark of acute myeloid (AML) and lymphoblastic (ALL) leukemia. This subgroup of leukemia exhibits a particular poor outcome, especially in infant.^3,4^ To date, 135 *KMT2A* rearrangements have been described; these chromosomal rearrangements give rise to oncofusion proteins that impair the differentiation of hematopoietic stem cells (HSCs) by epigenetic reprogramming.^5–9^

HOX genes are the prime targets of *KMT2A*-fusion products and regulate cellular differentiation in normal hematopoietic development.^5^ Despite the wide range of potential fusion partners, only 9 account for over 90% of the *KMT2A* rearrangements and the most frequently diagnosed are *KMT2A-AFF1* (*MLL-AF4*), *KMT2A-MLLT3* (*MLL-AF9*), *KMT2A-MLLT10* (*MLL-AF10*), and *KMT2A-MLLT1* (*MLL-ENL*).^8^

Analyses of neonatal blood spots, the blood collected by heel prick test, have suggested that *KMT2A-AFF1* translocations arise in utero and rapidly lead to the development of overt ALL, often at or shortly after birth.^10–12^ This argues in favor of the existence of preleukemic clones that do not need cooperating mutations to develop a malignant disease.^10,13^ In the last 20 years, several groups have investigated the role of cooperating mutations in the development, maintenance, and relapse of *KMT2A*-rearranged leukemia. The availability of the correct mouse models and the power of sequencing technologies have represented major challenges toward a unified working model.

Several murine mouse models have been developed to study *KMT2A*-rearranged leukemia in vivo. While the expression of some KMT2A fusion proteins is sufficient for the development of full blown AML in mice,^14–21^ the development of mouse models of ALL has proved more challenging. For example, *KMT2A-MLLT1* is frequently associated with B cell ALL in humans, but it generates AML in mice.^22^ In 2 studies, *KMT2A-AFF1* has shown to induce lymphoma in mice with a latency time of 12 to 14 months for the disease phenotype to become overt.^23,24^ Whereas the enforced expression of *KMT2A-AFF1* in human HSC does not induce leukemia, transduction of human HSCs with a *KMT2A-Aff1* construct carrying the human *KMT2A* fused to the murine *Aff1* does induce pro-B ALL within 22 weeks.^25^ Notably, in humans *KMT2A-AFF1* translocations give rise to ALL and are mostly found in infants younger than 1 year old.^8,9,12^

**Figure 1:**
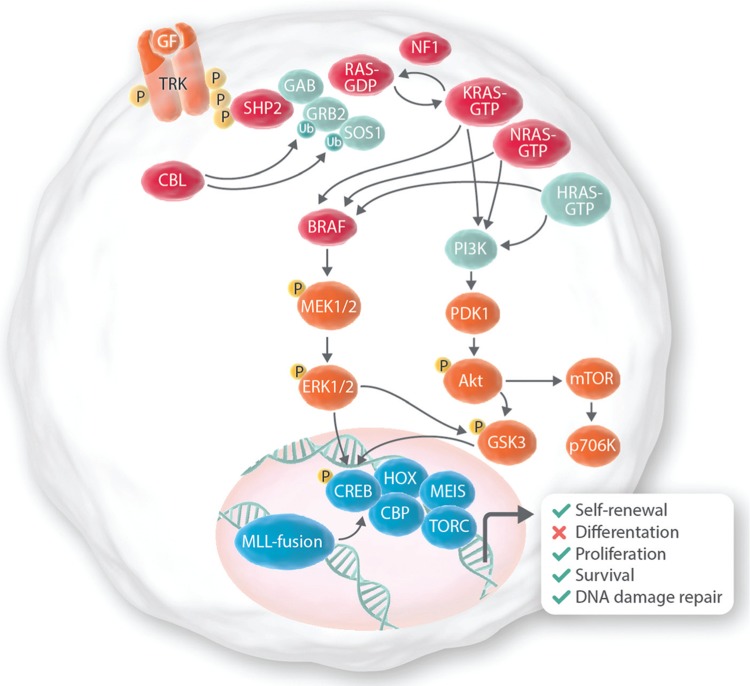
**The PI3K/Ras signaling pathway.** This illustration of the Ras signaling pathway highlights proteins affected by mutations in *KMT2A*-rearranged leukemia (pink). Growth factor (GF) binding to cell surface receptors results in activated tyrosine kinase receptor (TRK) complexes, which contain adaptors such as GRB2 (GF receptor bound protein 2) and Gab (GRB2-associated binding) proteins. Upon binding of a GF, such as G-CSF, to a receptor tyrosine kinases (RTKs), the RTK is autophosphorylated. This creates docking sites for adaptor molecules (eg, GRB2) and for the phosphatase SHP2. These molecules recruit and activate guanosin exchange factors (eg, SOS1) that catalyzes the GDP/GTP exchange on RAS. The GTPase-activating protein neurofibromin (NF1) binds to Ras-GTP and accelerates the conversion of Ras-GTP to Ras-GDP which terminates signaling. RAS-GTP activates several pathways and some are described here. The BRAF–mitogen-activated and extracellular signal-regulated kinase (MEK)–extracellular signal-regulated kinase (ERK) cascade primarily determines proliferation via activation of expression of genes important for cell cycle entry. RAF phosphorylates MEK1 and/or MEK2, which in turn activates ERK1 and/or ERK2. Activation of ERKs prompts a cascade of events that culminate in the activation of transcriptional regulators such as MYC, ELK1, FOS, ETS and expression of proteins regulating the cell cycle entry such as cyclin D1. This pathway also regulates the transcriptional activity of CREB/HOX/MEIS complex, which specifically regulates the proliferation and survival of *KMT2A*-leukemic cells via HOXA9 activity. Ras also activates the PI3K–3-phosphoinositide-dependent protein kinase 1 (PDK1)–Akt pathway that frequently determines cellular survival via inhibition of apoptosis. Active RAS is also able to activate PI3K/Akt pathway by a direct interaction with the PI3K p110 catalytic subunit. This leads to activation of downstream kinases such as PDK1 and AKT. AKT is a Ser/Thr kinase that promotes survival by phosphorylating, and therefore inactivating, several proapoptotic proteins such as BAD and BAX and regulators of cell cycle such as p27 and p21 and also regulates the activity of GSK3. PI3K = phosphatidylinositol 3-kinase.

In addition to the availability of the correct mouse models, another technical challenge is the limitation of sequencing technologies. Both solid and hematological malignancies are made of several subclones, this means that within a cancer there is a bulk of cancer cells sharing a subset of mutations, which are defined as clonal. From each of these clones, at every cell division, new subclones might emerge and they can carry new, additional mutations.^26^ Thus, although leukemia has a clonal origin, a leukemia is typically a collection of related but genetically distinct populations best considered to be subclones. This has important implications for treatment and clonal evolution of the disease. Recent studies have shown that somatic mutations affecting the kinase/phosphoinositide 3-kinase (PI3K)/RAS signaling pathway genes, including the PI3K catalytic α polypeptide, (*PI3KCA*), PI3K regulatory subunit α (*PI3KR1*), the GTPase activating protein (GAP) NF1 (*NF1*), the guanosine diphosphate (GDP)/guanosine triphospate (GTP) molecules *NRAS* and *KRAS*, the phosphatase SHP2 (*PTPN11*) and the ubiquitin ligase *c-CBL* are found in *KMT2A*-rearranged patients (Fig. 1).^27–43^ The PI3K/RAS pathway is activated in response to a variety of extracellular stimuli and transduce signals from the cell surface to the nuclear targets that play a key role in proliferation, apoptosis, and differentiation^44,45^ (Fig. 1). Although the scientific community has accumulated convincing evidences on the role of activated FLT3, which also signals through the Ras pathway, in the development of adult *KMT2A*-AML^2,40,46^ and FLT3 has been proven a therapeutic target in both AML and ALL,^47,48^ the role of PI3K-RAS pathway mutations on *KMT2A*-driven AML and ALL is controversial. This review summarizes the recent progress on our understanding of the role of mutations in PI3K/RAS pathway in initiation, maintenance, and relapse of *KMT2A*-rearranged leukemia.

## The detection of cooperating mutations; from the Sanger to Next-Generation Sequencing era

### *RAS* mutations

RAS proteins act as molecular switches by cycling between an active GTP-bound (RAS-GTP) and an inactive GDP-bound (RAS-GDP) conformation that regulate cell fates by coupling receptor activation to downstream effector pathways (Fig. 1). RAS proteins are encoded by 3 genes, *KRAS* (v-Ki-ras2 Kirsten rat sarcoma viral oncogene homolog), *NRAS* (neuroblastoma RAS viral [v-ras] oncogene homolog), and *HRAS*. The N-terminal portion of HRAS, KRAS, and NRAS comprises a highly conserved G domain that has a common structure.^45^ RAS proteins diverge substantially at the C-terminal end that specifies post-translational protein modifications. In most cases, the somatic missense *RAS* mutations found in cancer cells affect the aminoacid positions 12, 13, and 61. These changes impair the intrinsic GTPase activity of RAS and confer resistance to GAPs. Thereby mutant RAS proteins accumulate in the active, GTP-bound conformation. Constitutively active mutants of RAS induce oncogenic transformation through activation of the MAPK cascade^45^ (Fig. 1).

**Figure 2:**
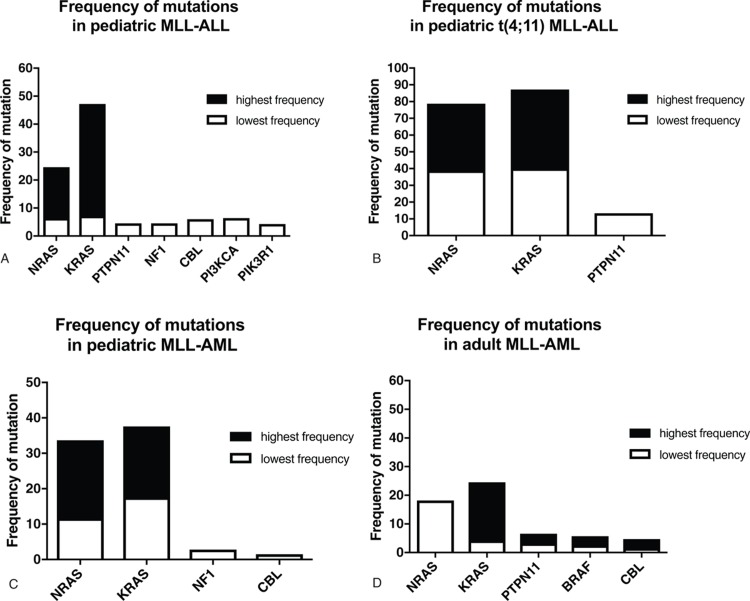
**Frequency of PI3K/RAS pathway mutations in *KMT2A-*driven leukemia.** The figure reports the lowest and the highest frequency of PI3K/RAS pathway mutations in *KMT2A-*driven leukemia in pediatric MLL-ALL (A), in pediatric MLL-ALL t(4;11) (B), in pediatric MLL-AML (C), and in adult MLL-AML (D). PI3K = phosphatidylinositol 3-kinase.

The first study investigating the presence of *RAS* mutations by Sanger Sequencing in *KMT2A*-rearranged ALL was published 20 years ago and found no *RAS* mutations in these patients.^27^ Subsequently independent groups have reported *RAS* mutations in up to 80% of *KMT2A*-rearranged ALL and 50% *KMT2A*-rearranged AML (Fig. 2 and Supplemental Table 1, Supplemental Digital Content).^27^*RAS* mutations were most common in infant with *KMT2A-AFF1*-rearranged ALL^28–30^ (Fig. 2). Whereas in adult *KMT2A*-rearranged AML, a statistical significant correlation between mutations and overall survival was not found,^34^*RAS* mutations, independently, contribute to a poor outcome in *KMT2A*-rearranged infant ALL patients.^29^ In the study by Driessen et al, *RAS* mutations were not present in the main leukemic clone but in subclones.^29^ This highlights that the frequency of these mutations might have been underestimated in the previous studies due to the fact that the Sanger Sequencing could not detect mutations with a very low mutant allele frequency. The advent of the Next-Generation Sequencing technologies has enabled the detection of rare subclonal mutations.^49,50^ Taking advantage of this technology Andersson et al performed a detailed genome and transcriptome wide analysis on diagnostic and matched relapse samples of infant *KMT2A*-rearranged ALL and found that although the genome of these leukemias are “silent,” with only 1.3 somatic mutations per clone, the most mutated pathway was the RAS/PI3K pathway.^31^ This has been confirmed by other studies^32,33,35,43^ (Fig. 2 and Supplemental Table 1, Supplemental Digital Content). Interestingly, in the majority of the cases, the mutations were subclonal.^31–33,35,43^ Clonal but not subclonal mutations showed a correlation with a worse outcome.^33^ In agreement with previous data,^29^ a higher frequency of *RAS* mutations was detected in infants rather than in older patients, corroborating the concept that aberrant *RAS* expression might shorten leukemia latency.

A further confirmation of the subclonality of these mutations came from the studies of matched diagnostic and relapsed samples. These analyses showed that these mutations might be lost,^30,31,33–35,43^ maintained/expanded,^30–34,36^ or gained at relapse.^33,42^

These studies highlight that *RAS* mutations are frequent at diagnosis (25–73% frequency) in patients carrying *KMT2A* rearrangements, in particular in pediatric *KMT2A-AFF1* cases, and that these mutations can be either clonal or subclonal secondary genetic events that might take part of a delicate selection process underlying clonal evolution of leukemia. Clonal but not subclonal mutations correlate with a worse outcome in *KMT2A-AFF1* ALL.

### *PTPN11* mutations

*PTPN11* encodes for the phosphatase SHP2, a ubiquitously expressed SH2 domain-containing protein tyrosine phosphatase (PTP), required for the full activation of the RAS–RAF–mitogen-activated and extracellular signal-regulated kinase (MEK)–extracellular signal-regulated kinase (ERK) pathway (Fig. 1). Constitutively active mutants of SHP2 induce oncogenic transformation through activation of the MAPK and PI3K/AKT cascade.^51–54^*PTPN11* mutations have been initially associated with the development of myeloproliferative disorders (MPD), juvenile myelomonocytic leukemia (JMML), and AML.^45,51,55–57^ More recently, a few studies have shown that single *Ptpn11* gain-of-function mutations predispose mice to acute leukemias^58,59^ suggesting that *PTPN11* mutations play a causal role in the development of this disease. More importantly, activating *PTPN11* mutations have been described in *KMT2A*-rearranged ALL^31,33^ and AML^34,36,40^ with a frequency of ca.3% for AML and 4% to 13% in ALL (Fig. 2 and Supplemental Table 1, Supplemental Digital Content). These are particularly high considering that *PTPN11* mutations have been found in 4% to 5% of overall AML patients and 6% of ALL patients.^60^

The most common mutations are located in the N-SH2 domain and include *PTPN11*^*D61Y*^, *PTPN11*^*A72V*^ and *PTPN11*^*E76K*^. These mutants exhibit higher tyrosine phosphatase activity. Mutations in the PTB domain including *PTPN11*^*S502T*^ and *PTPN11*^*G503A/R/L/E*^ have been reported in a case study and exhibit higher phosphatase activity as well.^18^ These mutants dephosphorylates yet unknown substrates that negatively affect RAS signaling. Analysis of matched diagnostic and relapsed samples showed that *PTPN11*^*G60V*^ were retained in pediatric ALL patient while *PTPN11*^*E76Q*^ were gained at relapse.^33^

### Mutations in other PI3K/RAS pathway genes

Few studies have reported mutations in other genes belonging to the PI3K/RAS pathway. BRAF is a member of the RAF kinase family^45,61^ (Fig. 1). Active RAS culminates in the activation of MAPK pathway via Ser/Thr kinase BRAF. BRAF phosphorylates MEK1 and/or MEK2, which in turn activates ERK1 and/or ERK2. *BRAF* mutations have been found in *KMT2A*-rearranged AML and ALL (Fig. 2 and Supplemental Table 1, Supplemental Digital Content).^29,34,36^ Most cancer-associated *BRAF* mutations encode gain-of-function mutants that constitutively activate the kinase and the MEK–ERK pathway. The mutations seem to disrupt the interaction of the glycine loop and activation segment, which destabilizes the inactive conformation of the protein.

*NF1* encodes neurofibromin, which is a GAP that inhibits RAS signaling by hydrolysis of active RAS-GTP into inactive RAS-GDP (Fig. 1). Therefore, NF1 deficiencies act as functional equivalents of activating mutations in RAS.^62^ Inactivating *NF1* mutations lead to the development of a human developmental disorder, the Neurofibromatosis 1, characterized by mental retardation, facial dysmorphism, and increased risk for developing malignant tumors, including JMML and AML.^63,64^ Few studies have also reported *NF1* deletions in *KMT2A*-rearranged leukemias (Fig. 2 and Supplemental Table 1, Supplemental Digital Content).^31,37^

*CBL* encodes an E3 ubiquitin ligase that negatively regulates receptor tyrosine kinase (RTK) and proteins regulating RAS activity, such as GRB2 and SOS (Fig. 1). Mutations in the linker region and RING finger domain of CBL impair the ability of Grb2 and SOS to suppress RAS signaling, and thereby over-activate RAS.^65^ CBL mutations have been found at high frequency (15%) in JMML and in 1% to 2% of adult AML.^66,67^ Recent studies also reported *CBL* mutations in *KMT2A*-rearranged patients (Fig. 2 and Supplemental Table 1, Supplemental Digital Content).^36,39,41^

Activating mutations in *PIK3CA* and *PIK3R1* have been reported in *KMT2A*-rearranged ALL by Andersson et al (Table 1).^31^

**Table 1:**
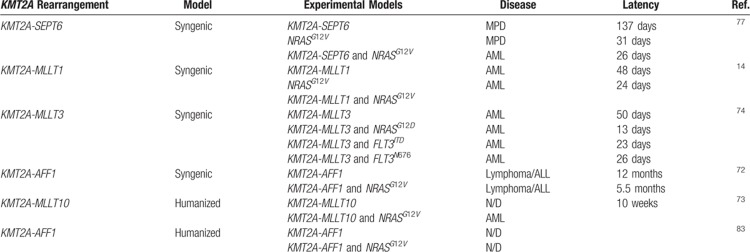
Summary of In Vivo Studies Addressing the Role of PI3K/RAS Pathway Mutations in *KMT2A-*driven Development

## Contribution of clonal cooperating mutations to *KMT2A*-driven leukemogenesis

Experimental mouse models have enabled to understand the role of cooperating mutations in initiation, maintenance, and relapse of leukemia. When modeled using murine systems, gain-of-function mutations in *Ptpn11*, *K-Ras*, and *N-Ras*, or loss-of-function in *Nf1* and *Cbl* are not sufficient to induce leukemogenesis but they induce cytokine hypersensitivity in myeloid progenitors and MPD in mice.^51,55,58,59,68–71^ Coexpression of these mutations with *KMT2A*-rearrangement has provided substantial evidence to the hypothesis that some cooperating mutations may synergistically cooperate with *KMT2A* rearrangements in leukemogenesis.^14,72–74^

### Syngenic mouse models of *KMT2A*-leukemia

The in vivo leukemogenesis assay is based on injection of mouse HSC that have been retrovirally transduced with a *KMT2A*-fusion gene, in syngenic mice lethally or sublethally irradiated. Serial transplantation of primary leukemia in secondary recipients allows to evaluate the impact of additional genetic and epigenetic events in the disease onset.^75^ However, these models are far from being perfect. The phenotype and pathogenesis of the leukemia developed in these murine models sometimes do not match those observed in human leukemia associated with the same genetic lesion, in part because of the biological differences between human and mouse HSCs.^76^

A controversial study by Ono et al concluded that *NRAS*^*G12V*^ cooperated with *KMT2A-SEPT6*. The mice receiving the HSC expressing *KMT2A-SEPT6* and *NRAS*^*G12V*^ died of AML with a significantly shorter latency of those receiving *KMT2A-SEPT6*, which instead died of MPD (26 ± 2.4 days vs 137 ± 9.0 days). However, in this setting, mice receiving *NRAS*^*G12V*^ alone died with a similar latency than those receiving *KMT2A-SEPT6* and *NRAS*^*G12V*^ (31 ± 1.4 days vs 26 ± 2.4 days), although they developed MPD rather than AML (Table 1).^77^

Zuber et al investigated the cooperation between *KMT2A*-rearrangement (*KMT2A-MLLT1*) and *RAS* mutation (*NRAS*^*G12V*^). They found that *NRAS*^*G12V*^ accelerated the disease onset in mice injected with HSC transduced with *KMT2A-MLLT1* (24 days vs 48 days) (Table 1).^14^ Hyrenius-Wittsten et al studied the effect of *NRAS*^*G12D*^, *FLT3*^*ITD*^, and *FLT3*^*N676K*^ on *KMT2A-MLLT3* leukemia development.^74^*FLT3* is a tyrosine kinase receptor overexpressed in *KMT2A*-rearranged leukemia and it is also commonly mutated in AML.^2,5,40^ Despite the fact that the percentage of cells coexpressing *KMT2A-MLLT3* and one of the cooperating mutations was very low (1–3%), the mice receiving cotransduced cells succumbed of leukemia much earlier than those receiving cells only transduced with *KMT2A-MLLT3* (median latency of 13, 23, 26 days, respectively, vs 50 days for *KMT2A-MLLT3* alone) (Table 1). These data indicate that these mutations, when occurring in the founder clone, can accelerate the onset of AML driven by *KMT2A-MLLT3*.^74^ The authors then performed a serial transplantation, by transplanting primary murine *KMT2A*-rearranged AML in secondary recipients. Interestingly, the disease latency of the secondary *KMT2A-MLLT3* mice was very similar to those of the secondary mice that coexpressed an activating mutation indicating that secondary *KMT2A-MLLT3* mice had accumulated additional genetic and epigenetic events that accelerated the disease onset. Further analysis of the bone marrow of these mice indicated a consistent activation of Akt, p38, and ERK pathways. These data therefore indicate that after serial transplantation the leukemic cells expressing *KMT2A-MLLT3* adapted in vivo to acquire a phenotype similar to those that coexpressed activating mutations. This suggest that these mutations might confer clonal fitness.^74^ Tamai et al developed a transgenic mouse carrying *KMT2A-AFF1* and *KRAS*^*G12V*^ that developed B-cell lymphoma/ALL in 6 months.^72^ Previous mouse models failed to recapitulate the human leukemia induced by *KMT2A-AFF1*.^20,23,24^ In the study of Tamai et al, mice developed lymphoma/ALL significantly earlier than the transgenic mice carrying *KMT2A-AFF1* alone (median survival 5.5 months vs 12 months), suggesting that *KRAS* mutations contributes to the early onset of ALL driven by *KMT2A-AFF1* (Table 1).^72^

Limited studies have addressed the contribution of *Ptpn11* to *KMT2A*-driven leukemia in vivo. As shown for RAS, coexpression of *Ptpn11*^*S506W*^ and *Ptpn11*^*E67k*^ accelerated *KMT2A-MLLT3* leukemia development in syngenic mouse models.^74,78^ In vitro studies have demonstrated that SH2 and PTB domains *PTPN11* mutants such as *PTPN11*^*E76K*^, *PTPN11*^*A72V*^, *PTPN11*^*D61Y*^, and *PTPN11*^*G503A*^ lose the autoinhibitory feedback, increased phosphatase activity, and induced hypersensitivity to IL-3 and GM-CSF. This can provide a growth advantage to the leukemic clones.^18,51,55,70,79,80^

Overall, these studies suggest that distinct *KMT2A* rearrangements have different dependence on *RAS* signaling or that a certain threshold of RAS signaling may be necessary to establish a leukemic disease in the murine models.

### Humanized mouse models of *KMT2A*-leukemia

Humanized mouse models have also attempted to clarify the role of cooperating mutations in *KMT2A*-rearranged leukemia. In these models, human cord blood-derived HSC are retro- or lentivirally transduced with a *KMT2A*-fusion gene and then transplanted into immunodeficient mice, such as NOD/SCID, that lack mature B and T cells, or NSG mice that lack mature NK, B, and T cells.^81^ Barabe et al showed that *KMT2A-MLLT1* and *KMT2A-MLLT3* induce AML in NOD/SCID mice in which the NK cells were depleted by an anti-NK cell antibody.^21^ Likewise Wei et al showed that *KMT2A-MLLT3* induces AML in NOD/SCID mice that express transgenic human SCF, GM-CSF, and IL-3 genes suggesting that human cytokines may provide the *KMT2A-MLLT3*-transdused HSCs with additional signals for cell growth and survival, acting as oncogenic promoters.^16^ Interestingly, although *KMT2A-MLLT10* does not induce leukemia on its own, mice transplanted with HSC expressing *KMT2A-MLLT10* and *KRAS*^*G12V*^ developed AML and died within 10 weeks from transplantation (Table 1).^73^ In this contest, expression of *KRAS*^*G12V*^ can mimic the signaling stimulated by the cytokines reported in the study by Wei et al since stimulation with SCF and IL-3 directly activates the RAS signaling pathway in leukemogenesis.^73^ By contrast, the enforced expression of *KMT2A-AFF1* in human HSCs does not induce leukemia,^82^ even in cooperation with *KRAS*^*G12V83*^. This is in contrast with the results obtained in the syngenic model.^72^ Whereas the inability to generate a faithful model of *KMT2A-AFF1* ALL supported the hypotheses that this oncogene could be unable to transform cells without cooperating oncogenes, the failure of models exploring the role of cooperating mutations^83–85^ have fuelled other hypotheses such as that *KMT2A* fusions might have a different dependence on *RAS* signaling,^73^ distinct *KMT2A* fusions might target different progenitor cells^82,86,87^ or strictly depend on the bone marrow microenvironment.^25,76^ These hypotheses are brilliantly reviewed by Sanjuan-Pla et al.^13^ Notably, the gene expression profile of *KMT2A-AFF1*-*KRAS*^*G12V*^ human HSC indicates an up-regulation of genes important in cell motility and cell migration and a down-regulation of genes important for cell adhesion, aggregation, and mesenchymal-epithelial transition.^30,88^ These data are in agreement with the Gene Ontology signature of *KMT2A-AFF1* patients. This would also explain the clinical hallmarks of *KMT2A-AFF1* patients who suffer from extramedullary hematopoiesis and central nervous system infiltration.

This suggests that clonal *RAS* mutations might be important in maintenance and chemotherapy resistance rather than initiation of *KMT2A-AFF1*-driven leukemia.

## The impact of subclonal mutation on *KMT2A*-driven leukemogenesis

All the in vivo studies reviewed in the previous paragraphs were based on human or mouse HSC cotransduced with retro- or lentivirus vectors expressing the *KMT2A*-rearrangement and the cooperating mutation of interest. These models enabled the authors only to address the effect of clonal mutations in the development of *KMT2A*-rearranged leukemia. Very limited information is available on the impact of subclonal mutations on *KMT2A*-driven leukemogenesis. A recent study from Hyrenius-Wittsten et al elegantly addresses how subclonal mutations affecting *FLT3*^*N676K*^ contribute to the development of *KMT2A-MLLT3* leukemia. The authors injected cells expressing *KMT2A-MLLT3* and the activating mutation *FLT3*^*N676K*^ and cells expressing only *KMT2A-MLLT3* at various ratios (from 1:28 to 1:156). Despite the low number of cells coexpressing *KMT2A-MLLT3* and *FLT3*^*N676K*^, mice receiving cotransduced cells succumbed of leukemia much earlier than those receiving cells only transduced with *KMT2A-MLLT3* (median latency 34 days vs 50 days for *KMT2A-MLLT3* alone), confirming that subclonal activating mutations in *FLT3* accelerate *KMT2A-MLLT3* leukemogenesis Hyrenius-Wittsten.^74^ Disease latency in secondary recipients was just slightly different (median latency 16 days vs 21 days for *KMT2A-MLLT3* alone). As the *FLT3*^*N676K*^ construct was fused with GFP, the authors could analyze the clone size by fluorescence. The data indicate that in most of the mice the clone increased in size; however, there were also cases where the clone was maintained or lost. This, together with the shorter latency of mice receiving *KMT2A-MLLT3* and *FLT3*^*N676K*^, suggests that the clone might have acquired de novo mutations to adapt in vivo. Sequencing revealed that the secondary recipients show de novo mutations in PI3K/RAS pathway genes such as *Ptpn11*, *Cbl*, *Braf*, and *Kras*. This suggests that the subclones *KMT2A-MLLT3+FLT3*^*N676K*^ might influence the environment and increase the chances for other clones to acquire specific mutations. The authors investigated the expression of cytokines and growth factors and found that several genes were differentially regulated in the leukemic cells. One commonly upregulated in cells expressing *KMT2A-MLLT3* and a cooperating mutation (either *NRAS*^*G12D*^, *FLT3*^*ITD*^, or *FLT3*^*N676K*^) but not in cells expressing *KMT2A-MLLT3* alone and normal healthy hematopoietic subpopulations, was the macrophage Migration Inhibitory factor (*Mif*).^74^ These data agree with previous data reporting a marked increase in inflammatory cytokines IL-1β, CCL12, CCL4, in the plasma of transgenic mice *Ptpn11*^*E76K*^*/+Mx1-Cre+* mice, where the *Ptpn11*^*E76K*^ mutation is expressed in HSC and bone marrow microenvironment.^58^ Thus, it appears that persistent high levels of proinflammatory cytokines hyperactivate the bone marrow microenvironment and displace HSC from their niches, contributing to the development of MPD that might lead to AML.^58^ The results of Hyrenius-Wittsten et al highlight the importance of *FLT3*^*N676K*^ in the initiation and in the progression of *KMT2A-MLLT3* leukemia and suggest that cooperating mutations might facilitate an inflammatory environment that could play an important role on the selection process underlying clonal evolution of leukemia.

## Are mutations in PI3K/RAS pathway second hits for early *KMT2A*-leukemia onset?

The studies reviewed so far highlight that mutations in the PI3K/RAS pathway are frequent at diagnosis in patients carrying *KMT2A* rearrangements.

It is difficult to establish the role of these mutations in pediatric versus adult *KMT2A*-driven ALL because only one of the studies reviewed here reports the mutation frequency in *KMT2A*-rearranged adult ALL. In this study, by Prelle et al, the frequency of *RAS* mutations is higher in pediatric than adult patients (26% vs 8%).^30^ Nevertheless, the authors neglect that these additional mutations might be the second hits for early onset of *KMT2A-AFF1* ALL. The authors disagree with this speculation because the frequency of the mutations is not sufficient to fully sustain the 2-hit model. If we sum the frequency of all the mutations reported in the PI3K-RAS pathway in each study, we can clearly observe that in pediatric *KMT2A-AFF1* cases the mutation frequency reaches 80% to 90%, at least in the most recent publications where more sensitive sequencing technologies have been applied.^32,33^ This can lead us to speculate that mutations in the RAS/PI3K pathway could be the second hits for early leukemia onset at least for some patients, as those carrying the translocations *KMT2A-AFF1*. However, the subclonality of these mutations, the fact that they are often lost at relapse and the lack of a robust in vivo model do not fully fit with this hypothesis.

Additional studies are required to determine whether in the cases with *KMT2A*-rearrangements without PI3K/RAS mutations or in the cases where the mutations are subclonal there are alternative modes of RAS pathway activation. For example, in chronic myeloid leukemia, where *RAS* mutations generally are absent, the RAS signaling pathway is deregulated by the BCR-ABL fusion protein.^89^ In view of this, it is interesting that both *KMT2A-AFF1* and *KMT2A-MLLT3* have been shown to directly activate RAS pathway through activation of ELK-1 protein, a downstream target of the RAS/RAF signaling pathway.^90,91^ In addition, KMT2A-AF6 has been shown to aberrantly activate RAS and its downstream signaling.^92^ Thus, these fusion proteins are per se able to activate the RAS/RAF signaling cascade by themselves and, therefore, *RAS* clonal mutations might actually not be necessary. Unfortunately appropriate in vitro and in vivo models are not yet available to fully elucidate the role of RAS activation in *KMT2A*-leukemogenesis. It is, however, notable that *RAS* mutations are a predictor of poor outcome in *KMT2A*-rearranged pediatric ALL and therefore this information can help to stratify patients.^29^

Mutations in *NRAS* and *KRAS* are more frequent at diagnosis in *KMT2A*-rearranged AML than *KMT2A*-rearranged ALL (Fig. 2). In the majority of the AML cases, the subclones with RAS pathway mutations detected at diagnosis are not detectable at relapse. An exception to this pattern is mutations in *KRAS* that were instead found expanded in relapse samples.^34,36^ However, in contrast to infant *RAS*-mutant *KMT2A*-rearranged ALL, the presence of these mutations does not impact the outcome of the patients. Again, in contrast to infant *RAS*-mutant *KMT2A*-rearranged ALL where the contribution of RAS activation is still under debate, several studies have proven that RAS activation and activation of RAS downstream targets, MAPK and PI3K signaling, confers a proliferative advantage to AML leukemic cells and is almost ubiquitous in AML.^77,93^ In addition to the known somatic activating mutations in *RAS* and RAS activating genes *NF1* and *PTPN11*, that we have reviewed here, constitutive activation of several RTKs, such as colony stimulating factor 1 and FLT3, which also partially signals through the RAS pathway, contribute to the pathogenesis of AML.^5,46^ Together, these observations indicate that RAS mutations and/or activation provide a growth advantage to *KMT2A*-AML cells and have therefore fuelled intense interest in the development of RAS-targeted AML therapy as described below.

## Targeting the RAS pathway in *KMT2A*-driven leukemia

The high incidence of RAS pathway mutations at diagnosis, their abundance in the major clone of relapsed leukemic cells and association with high-risk disease, provide a rationale to investigate potential novel therapies that exploit pathway activation, such as kinase inhibitors that act downstream of RAS targeting the RAF/MAPK or PI3K/AKT pathway or farnesyltransferase inhibitors preventing the post-translational modification of RAS, in *KMT2A* patients.^94,95^ MEK1/2 inhibitors are the most promising as ERK1/2 are the only downstream targets of MEK1/2, whereas ERK can phosphorylate over 150 targets.^96^ MEK1/2 inhibitors, such as Selumetinib, Pimasertib, and Trametinib, are in clinical trials and have moderate efficacy as single agents in AML and ALL, particularly in leukemias harboring RAS pathway mutations.^42,97–101^ A few studies have addressed their efficacy in *KMT2A*-rearranged AML and ALL.

Kerstjens et al investigated the effect of MEK inhibitors in infant *KMT2A*-ALL carrying *RAS* mutations and found that *RAS* mutant *KMT2A*-ALL cell lines and primary samples are sensitive to Trametinib, MEK162, and Selumetinib, whereas non-*RAS* mutant *KMT2A*-ALL cell lines and primary samples were insensitive to MEK1 inhibitors.^102^ Interestingly, MEK inhibitors increased apoptosis and enhanced the sensitivity of *KMT2A*-ALL cells to prednisolone, regardless of the *RAS* mutational status, in agreement with previously published data.^100^ However, when tested in vivo, MEK inhibitor Trametinib did not inhibit leukemia progression in a *KMT2A*-rearranged ALL in vivo model.^103^ Trentin et al tested PD0325901, a potent inhibitor of MEK1/2 currently in phase II clinical trials, on *NRAS*^*Q61K*^ mutated *KMT2A-AFF1* B cell line MI04. The inhibitor reduced the proliferation of MI04 of 40% even at nanomolar concentrations, whereas the 2 *RAS* wild-type *KMT2A-AFF1* cell lines showed only a modest proliferative reduction even increasing the drug concentration.^32^ Importantly, in 1 *RAS* wild-type non-*KMT2A*-rearranged ALL patient Trametinib had an antagonistic effect on prednisolone sensitivity.^100^ This is in contrast with the data published by Jones et al that reported an agonistic effect between Trametinib and prednisolone in 2 non-*KMT2A* rearranged ALL patients who did not carry *RAS* mutations.^97^ Notably the study by Jones et al reported the agonistic effect in 3 out of 5 patients, and only one of them carried *RAS* mutations. These findings warn against a broad use of MEK inhibitors and suggest that the implementation of these inhibitors needs to be guided by relevant biomarkers (eg, *RAS* mutation) and preclinical in vitro evidence.

Although in AML no major differences between the transcriptomes of *KMT2A*-rearranged *RAS* wild-type and *KMT2A*-rearranged *RAS*-mutant cells have been found, kinome, proteome profiling, and chemical analyses revealed that the responses of these cells to MEK inhibitors were distinct, suggesting that MEK inhibitors could be effective.^36,104^

MEK inhibitor U0126 effectively reduced the cell survival of primary *KMT2A*-rearranged AML samples in contrast to normal bone marrow cells.^104^ Among the samples tested by Kampen et al, the ones carrying *RAS* mutations showed greater sensitivity to MEK inhibitors. Kampen et al also tested a range of AML cell lines carrying *NRAS* mutations and/or *KMT2A* rearrangement. The authors found that THP1 (*NRAS* mutated—*KMT2A* rearranged) showed greater sensitivity to MEK inhibitors than HL60 and OCI-AML3 (*NRAS* mutated—non-*KMT2A* rearranged) and *RAS* wild-type non-*KMT2A*-rearranged AML cell lines. By contrast, neither knockdown nor knockout of MEK1 affected the proliferation of THP1.^105^ These results might suggest that simultaneous targeting of MEK1 and MEK2 might be fundamental to achieve a therapeutic effect.

Despite the encouraging preclinical data obtained with MEK inhibitors on *KMT2A*-rearranged AML and ALL, a monotherapy is unlikely to be successful. In vivo data and phase II clinical trials showed that MEK inhibition only resulted in nonsustained responses, suggesting aberrant feedback mechanisms.^98^ Kampen et al showed in response to MEK inhibitors a sustained activation of AKT/mTOR and VEGFR-2 pathways that enabled a subpopulation of *KMT2A*-rearranged AML cells to survive MEK inhibition, suggesting the need to target multiple pathways.^104^ In agreement, Lavallée et al showed increased efficacy in *KMT2A*-rearranged *RAS* mutant AML samples when MEK inhibitors were used in combinations with VEGFR-2 inhibitors.^36^ Notably both *KMT2A-AFF1* and *KMT2A-MLLT3* have been shown to directly activate RAS pathway.^90,91^ This suggests that *KMT2A* patients might benefit from inhibitor of this pathway even if they do not carry *RAS* mutations. This hypothesis needs to be experimentally validated.

The identification of *PTPN11* mutations in *KMT2A*-rearranged leukemia and the requirement for SHP2 function in the RAS signaling pathway strongly suggest that small molecule SHP2 inhibitors might have important therapeutic applications. Indeed, SHP2 plays a key role in controlling apoptosis and the suppression of SHP2 expression induces differentiation, apoptosis and inhibits leukemic cell clonogenic growth.^79,80,106^ Unfortunately, the development of bioavailable SHP2 inhibitors has proven to be difficult. The discovery of SHP2-specific inhibitors is complicated by the extremely high homology of the catalytic core of SHP2 with those of SHP1 and other PTPs that play negative roles in growth factor and cytokine signaling. Yu et al characterized the inhibitor No. 220 to 324 in cell line and JMML primary samples and found that the drug effectively inhibited SHP2 activity and IL-3-induced ERK, AKT, and STAT5 activation in a dose-dependent manner.^107^

Other agents such as MEK and mTOR inhibitors might prove efficacious in *RAS*-mutated leukemias. Liu et al demonstrated the preclinical therapeutic efficacy of mTOR inhibitor Rapamycin in the mouse model of *Ptpn11* mutation-induced MPN and also in *PTPN11* mutation-positive JMML patient cells.^108^ Likewise Parkin et al showed that AML blasts that do not express NF1 display differential sensitivity to in vitro treatment with Rapamycin.^64^

## Conclusions and perspectives

The high frequency of activating mutations in tyrosine kinase/PI3K/RAS pathway genes in *KMT2A*-rearranged leukemia underscores the biologic cooperativity between KMT2A fusion proteins and signaling through this pathway. Experimental models have proved that some *KMT2A* rearrangements synergistically cooperate with *RAS* mutations in leukemogenesis, suggesting that these mutations could be important in the initiation of the disease. The controversial data on the crosstalk between *RAS* mutations and *KMT2A-AFF1* suggest that *RAS* mutations are not important for initiation of leukemia. Analyses of several diagnostic and relapse matched samples will be essential to address the contribution of these mutations to maintenance and relapse of *KMT2A*-rearranged leukemia. The mutational burden could also inform therapeutic decisions and indicate which patients could benefit from a targeted therapy. One can argue that targeting minor clones rather than the dominant one is a questionable approach; however, the experimental data indicate that *RAS* and *PTPN11* clonal mutations are associated with chemotherapy resistance and that MEK inhibitors enhance the sensitivity to prednisolone of both *RAS* wild-type and mutant ALL.^33,97,100,102,109^ Therefore, given that effective targeted therapies are currently not available for *KMT2A*-rearranged leukemia patients, these findings have important therapeutic implications.

## Supplementary Material

Supplemental Digital Content
