# Supplementary material for: The Impact of PI3-kinase/RAS Pathway Cooperating Mutations in the Evolution of KMT2A-rearranged Leukemia
Source: Hemasphere. 2019 Apr 17;3(3):e195. doi: 10.1097/HS9.0000000000000195 (PMC6746018; doi:10.1097/HS9.0000000000000195)
Supplement: Supplemental Digital Content [file hs9-3-e195-s001.docx]

**Supp Table 1 Frequency of PI3K/RAS pathway mutations in *KMT2A-*driven leukemia**

| **Leukemia type** | ***NRAS*** | ***KRAS*** | ***PTPN11*** | ***BRAF*** | ***NF1*** | ***CBL*** | ***PIK3CA*** | ***PIK3R1*** | **Ref.** |
| --- | --- | --- | --- | --- | --- | --- | --- | --- | --- |
| ALL  Pediatric  n=13 | 0% | 0% |  |  |  |  |  |  | ^27^ |
| ALL  Pediatric  n=20 | 10% | 40% |  |  |  |  |  |  | ^28^ |
| ALL  Pediatric  n=109 | 6.4%  * | 7.2%  * |  | 0% |  |  |  |  | ^29^ |
| ALL  Pediatric (not including t4;11)  n=38 | 2.6% | 7.9% |  |  |  |  |  |  | ^30^ |
| ALL  Peditaric (only t4;11)  n=31 | 22.5% | 3.2% |  |  |  |  |  |  | ^30^ |
| ALL  Adult (only t4;11)  n=48 | 4.16% | 4.16% |  |  |  |  |  |  | ^30^ |
| ALL  Pediatric  n=22 (+25 validation cohort) | 18.2% | 9.1% | 4.5% |  | 4.5% |  | 6.4% | 4.3% | ^31^ |
| ALL  Pediatric  n=92 | 7.6% | 14.1% |  |  |  |  |  |  | ^35^ |
| ALL  Pediatric (t4;11)  n=36 | 38.8% | 47.2% |  |  |  |  |  |  | ^32^ |
| ALL  Pediatric (t4;11)  n=15 | 40% | 40% | 13.3% |  |  |  |  |  | ^33^ |
| ALL  Pediatric  n=33 |  |  |  |  |  | 6% |  |  | ^41^ |
| ALL  Pediatric (t4;11)  (n=18) |  |  |  |  |  | 0% |  |  | ^39^ |
| AML  Pediatric  (n=10) | 0 | 20% |  |  |  |  |  |  | ^27^ |
| AML  Pediatric  n=17 | 11.76% | 17.6% |  |  |  |  |  |  | ^28^ |
| AML  Adult  n=118 | 22% | 20.3% | 3.4% | 2.5% |  |  |  |  | ^34^ |
| AML  Adult  n=33 | 18.2% | 42.4% |  |  |  |  |  |  | ^38^ |
| AML  Adult  n=31 | 32% | 16% | 3.2% | 3.2% |  | 3.2% |  |  | ^36^ |
| AML  Pediatric  n=71 |  |  |  |  | 2.8% |  |  |  | ^37^ |
| AML  Pediatric and adult  (n=64) |  |  |  |  |  | 1.5% |  |  | ^39^ |

*The frequency of *RAS* mutations is higher in the *KMT2A-AFF1* subgroup compared to the rest of *KMT2A*-rearranged ALL patients
